# Subclinical Dysfunction of Left Atrial Compliance after Cryoballoon versus Radiofrequency Ablation for Paroxysmal Atrial Fibrillation

**DOI:** 10.3390/jcm12154974

**Published:** 2023-07-28

**Authors:** Ewa Pilichowska-Paszkiet, Agnieszka Sikorska, Ilona Kowalik, Krzysztof Smarż, Małgorzata Sikora-Frąc, Jakub Baran, Roman Piotrowski, Tomasz Kryński, Piotr Kułakowski, Beata Zaborska

**Affiliations:** 1 Department of Cardiology, Centre of Postgraduate Medical Education, Grochowski Hospital, 04-073 Warsaw, Poland; asikorska@cmkp.edu.pl (A.S.); msikora@cmkp.edu.pl (M.S.-F.); jbaran@cmkp.edu.pl (J.B.); rpiotrowski@cmkp.edu.pl (R.P.); tkrynski@cmkp.edu.pl (T.K.); pkulakowski@cmkp.edu.pl (P.K.); bzaborska@cmkp.edu.pl (B.Z.); 2 Clinical Research Support Center, National Institute of Cardiology, 04-073 Warsaw, Poland; ikowalik@ikard.pl

**Keywords:** left atrial function, strain, cryoablation, atrial fibrillation

## Abstract

It has been suggested that cryoballoon (CB) ablation for paroxysmal atrial fibrillation (PAF) may lead to more extensive left atrial (LA) injury than radiofrequency (RF) ablation; however, results are conflicting. We sought to address this issue using modern echocardiographic techniques estimating the LA function after successful CB and RF ablation for PAF. A total of 90 patients (66% males, mean age 57 ± 10 years) successfully treated (no AF recurrences confirmed in serial 4–7 day ECG Holter monitoring) with RF (51%) or CB (49%) ablation for PAF were retrospectively studied. Echocardiography with speckle tracking (STE) was performed before and 12 months after the procedure. The peak longitudinal LA strain (LAS) and strain rate (LASR) during the reservoir (r), conduit (cd), and contraction (ct) phases were measured in sinus rhythm. Analysis of covariance was applied to compare changes in the echocardiographic parameters over time with the baseline measurements as covariance and the type of ablation as the factor. The parallelism of the slopes of the covariance was tested. The LA diameter decreased (38.3 ± 4.1 mm vs. 36.8 ± 3.6 mm, *p* < 0.001) in the whole study group at 12 months after ablation. The LASRr and LASRcd increased (1.1 ± 0.3 s^−^¹ vs. 1.3 ± 0.3 s^−^¹, *p* < 0.001 and 1.1 ± 0.3 s^−^¹ vs. 1.2 ± 0.3 s^−^¹, *p* < 0.001, respectively) whereas other LA strain parameters remained unchanged in the whole study group at 12 months after ablation. In the analysis of LA function at 12 months after the procedure regarding the mode of ablation, the worsening of parameters reflecting LA compliance was observed in patients with better pre-served baseline values in the CB ablation subgroup. For baseline LAScd >28%, the difference ΔCB − ΔRF was −7.6 (11.7; −3.4), *p* < 0.001, and for baseline LAScd >16%, ΔCB − ΔRF was −1.8 (−3.2; −0.4), *p* = 0.014. The traditional Doppler-derived parameter e′ showed the same trend—for baseline e′ ≥12 cm/s, ΔCB − ΔRF was −1.7 (−2.8; −0.6), *p* = 0.003. We conclude that worsening of parameters reflecting LA compliance was observed 12 months after CB ablation compared to RF ablation for PAF in patients who underwent a successful procedure and had better-preserved baseline LA function. This might suggest subclinical dysfunction of LA after the CB ablation procedure. The clinical significance of these findings warrants further investigations.

## 1. Introduction

Atrial fibrillation (AF) is associated with left atrial (LA) remodeling and fibrosis which deteriorate LA function [1]. Echocardiography with the use of speckle tracking (STE) analysis enables precise estimation of the LA function [2]. Catheter ablation for AF with pulmonary vein isolation (PVI) is an established tool in AF treatment; nonetheless, the procedure is associated with injury of atrial myocardium [3]. The ablation-related injury can lead to loss of cardiomyocytes with replacement fibrosis and in consequence impair LA function, especially in patients with pre-existing LA fibrosis [4]. Pulmonary vein isolation can be achieved by radiofrequency (RF) ablation using thermal heating or cryoballoon (CB) ablation using cryogenic freezing of the tissue with comparable rates of long-term success for both methods [5]. The mechanism of ablation-induced tissue damage is different in RF and CB techniques [5]. It has been suggested that CB ablation for paroxysmal AF may lead to more extensive LA injury than RF ablation; however, results are conflicting [6,7,8]. The aim of our study was to estimate the effect of ablation considering RF and CB techniques on LA function in patients with paroxysmal AF successfully treated with ablation.

## 2. Materials and Methods

### 2.1. Study Population

We retrospectively studied 90 patients with paroxysmal AF admitted to our institution for ablation. Echocardiographic images were recorded in a prospective manner for further analysis by investigators blinded to the mode of ablation. Patients were included in the two previously published studies [9,10] which assessed clinical and echocardiographic predictors of successful ablation.

**Figure 1:**
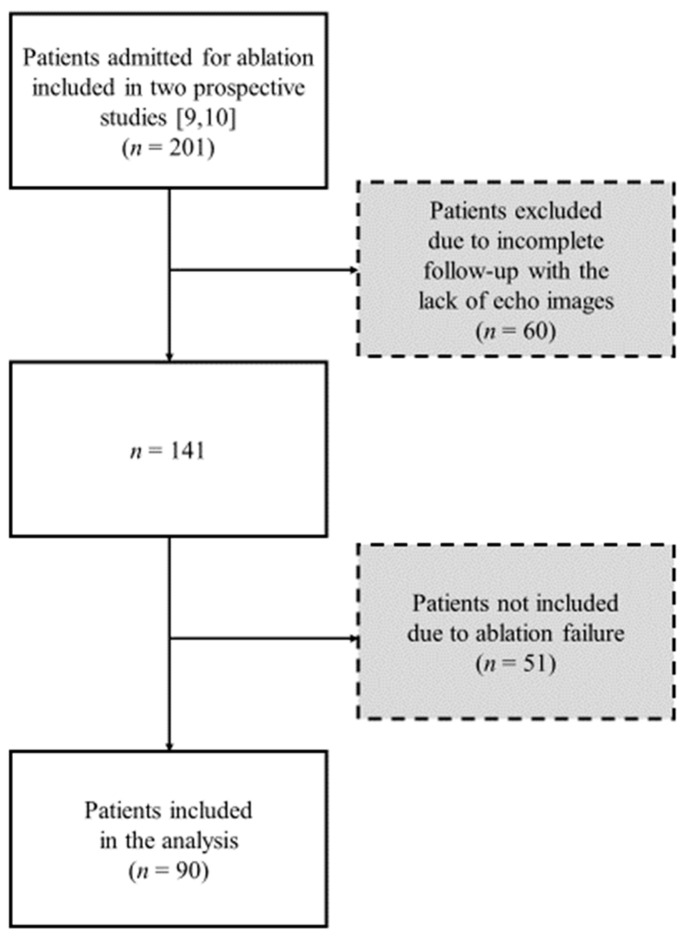
Flowchart of the studied population [9,10].

The inclusion criteria in both studies were as follows: paroxysmal AF without structural heart disease and first-time ablation. In order to be included in the present analysis, patients had to be in sinus rhythm before and after ablation, with successful procedure (freedom from the recurrence of arrhythmia, defined as AF or atrial tachycardia that lasted at least 30 s and was documented on standard ECG or during serial Holter ECG monitoring, without taking into account early recurrences corresponding to the blanking period of the first 3 months after the CA) and have complete follow-up. Data of 90 patients were complete and available for the analysis. Figure 1 shows the flowchart with the analysis of the patient population from which the study group was separated.

The studies were approved by the local ethics committee (approval number 58/PW/2011 and 65/PB/2015). All patients gave written informed consent to participate in the study.

### 2.2. Echocardiography

All patients underwent transthoracic echocardiography (TTE) within 24–48 h before ablation and 12 months after the procedure. TTE was performed using Vivid 9 (GE Medical System) by investigators blinded to the mode of ablation. Images were recorded and saved in the archive (EchoPac, GE Healthcare) for further analysis.

The cardiac dimensions were measured in accordance with the current recommendations [11]. The LA diameter (LAd) was measured at end-systole in the parasternal long-axis view. The LA volume (LAV) was calculated from the apical 4-chamber (4C) and 2-chamber (2C) views using biplane area–length method. The LAV index was defined as the LAV divided by the body surface area (BSA). Mitral flow velocities (E and A) were assessed by pulsed-wave Doppler (PW). Tissue Doppler imaging (TDI) was used to measure velocities of the early (e′) and late (a′) diastolic phases at the mitral annular septal and lateral corners. The E/e′ ratio was calculated by dividing E by the average of the septal and lateral e′ velocities.

**Figure 2:**
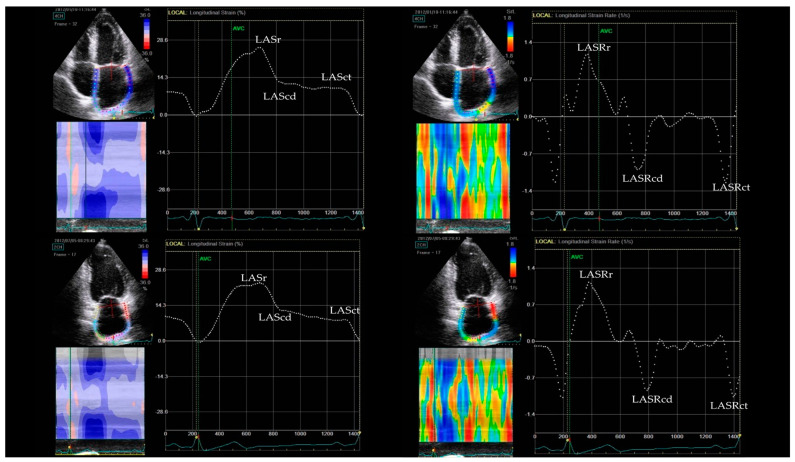
Measurements of LA strain in 4- and 2-chamber views (**left** panel) and LA strain rate in 4- and 2-chamber views (**right** panel). LASr—left atrial reservoir strain, LAScd—left atrial conduit strain, LASct—left atrial contractile strain, LASRr—left atrial reservoir strain rate, LASRcd—left atrial conduit strain rate, LASRrct—left atrial contractile strain rate.

Peak longitudinal LA strain (LAS) and strain rate (LASR) during the reservoir (r), conduit (cd), and contraction (ct) phases were measured by STE (Figure 2). All LAS measurements were analyzed according to the recent consensus document of the EACVI/ASE/Industry Task Force to standardize deformation imaging [12]. The images in the apical 4C and 2C views images were obtained with a frame rate set between 60 and 80 frames per second. Loops of 3 cardiac cycles were stored digitally and analyzed offline with software (EchoPac, GE Healthcare) by an experienced echocardiographer blinded to the mode of ablation. The LA endocardium was manually traced in the 4C and 2C views to create a region of interest (ROI) composed of six segments in each view. After segmental tracking quality analysis with the possibility of manual adjustments to the ROI, the software generated strain curves for each atrial segment. The global LAS for each phase was calculated by averaging the values observed in all LA segments. The zero-strain point was set at LV end-diastole. The LA stiffness index, the ratio of E/e′ to LASr, was calculated [13]. 

### 2.3. Catheter Ablation Procedure 

Patients underwent ablation performed according to widely accepted protocols [14]. Allocation to RF or CB ablation was random unless patients had a prominent common trunk of the left pulmonary vein assessed by cardiac computed tomography or intracardiac echocardiography. In such a case, patients were treated with RF ablation (*n* = 10). Point-by-point PVI using RF energy was performed after double transseptal puncture using irrigated ablation catheters (Thermocool SF or Thermocool SmartTouch ST), a LASSO catheter, and the CARTO 3 system (Biosense Webster, Los Angeles, CA, USA). Ipsilateral veins were isolated together with additional applications between the veins. The energy settings were 30 watts in the anterior wall, 20 to 25 in the posterior wall for 30 s (Thermocool SF catheter) or to achieve ablation index 500 and 350–400, respectively (Thermocool SmartTouch ST catheter).Cryoballoon PVI was performed using a single transseptal puncture. A steerable 15 Fr sheath (FlexCath Advance, Medtronic, Minneapolis, MN, USA) was positioned in the left atrium and an inner lumen mapping catheter for PV potential recordings (Achieve, Medtronic, Minneapolis, MN, USA) was advanced in each PV ostium. A 28 mm CB (Arctic Front or Arctic Front Advance, Medtronic, Minneapolis, MN, USA) was used. 

### 2.4. Follow-Up

The follow-up lasted one year. Patients were seen in the outpatient clinic 3, 6, and 12 months after ablation and underwent serial 4–7-day Holter ECG monitoring (DMS 300-4A, DM Software, Stateline, NV, USA). TTE was performed at 6- and 12-months visit. Pharmacological treatment was at the discretion of the attending cardiologist and was not part of the protocol.

### 2.5. Statistical Analysis 

Continuous and normally distributed variables were expressed as the mean ± standard deviation, continuous and non-normal distribution were reported by median and quartiles (25th percentiles, 75th percentiles), and categorical data were presented as counts and percentage. The normality of the distribution of continuous variables was tested with the Kolmogorov–Smirnov test. Baseline characteristics were compared using Student’s t-test for independent samples, Wilcoxon two-sample test, and X² test or Exact Fisher test, as appropriate. The paired Student’s t-test was used to compare echocardiographic parameters within the group and analysis of covariance was applied to compare their changes over time with the baseline measurement as covariance and the type of ablation as the factor. The parallelism of the slopes of the covariance was tested. Differences between the 12-month and baseline measurements were reported as means (or baseline-adjusted means) with 95% confidence interval. All analyses were conducted using SAS version 9.4 (SAS Inc., Cary, NC, USA), and a *p*-value < 0.05 was considered statistically significant.

## 3. Results

### Patient Characteristics

The study group consisted of 90 patients (65.6% males, mean age 57.2 ± 9.7 years) with paroxysmal AF who were successfully treated with ablation. A total of 46 (51%) patients underwent RF and 44 (49%) patients underwent CB ablation. There were no significant differences in baseline clinical characteristics between subgroups except greater B-blockers treatment in the RF subgroup compared to the CB subgroup—36 pts (78.3%) vs. 19 pts (44.2%), *p* = 0.001. The treatment was left to the discretion of the attending cardiologist and was not part of the protocol. Table 1 shows the baseline clinical characteristic of the study group.

There were no significant differences in baseline echocardiographic parameters between subgroups except higher LASRr in RF compared to CB ablation subgroup ( 1.20 ± 0.23 s^−^¹ vs. 1.09 ± 0.27 s^−^¹, *p* = 0.041).

Table 2 shows echocardiographic parameters at baseline and after 12 months of follow-up in the whole study group. The diameters of the left ventricle and the left atrium diminished. The velocities of the early (e′) and late (a′) diastolic phases at the mitral annular corners increased. There were no changes in the LA strain parameters at reservoir, conduit, and contraction phases of the LA cycle. The improvement of LASR during reservoir and conduit and stable contraction LA cycle were observed in the whole study group. 

Parameters of LA geometry and function regarding the mode of ablation at 12 months after the procedure are shown in Table 3. The changes in LA dimension did not differ significantly between the CB and RF subgroups. The significant improvement of the a′ velocity was observed in the CB subgroup, but the change in this parameter did not differ significantly between the CB and RF subgroups. The changes in LASR during reservoir and conduit LA phase did not differ significantly between subgroups.

Table 4 shows parameters, for which the differences between RF and CB subgroup depended on the baseline values (interaction effect was observed). A significant decrease in the velocity of the e′ and the value of LAS at conduit phase were observed in the CB compared to the RF ablation subgroup in patients with better preserved baseline values of these parameters (≥12 cm/s and ≥16%, respectively).

For changes in other analyzed echocardiographic parameters no interaction effect was found. The difference between RF and CB subgroups did not depend on the baseline values of these parameters.

## 4. Discussion

In our study, we found worsening of parameters reflecting LA compliance at 12 months after successful CB vs. RF ablation for PAF. This might suggest that CB and RF ablation differentially influence long-term LA function and subclinical dysfunction of left atrium after CB procedure can be present.,

The LA relaxation, chamber stiffness, and contractility influence reservoir, conduit, and contractile function, respectively [15]. The LA conduit phase characterizes LA function during early diastole when the mitral valve opens and the left atrium empties to the left ventricle [16]. The left atrium in this phase contributes to LV filling in early diastole by downstream suction of the blood within the “single” chamber formed by the left atrium and left ventricle during opened mitral valve. The LA conduit is influenced by LV diastolic properties and the pulmonary venous compartment. In the phase of passive atrial emptying, PVs drain blood from the lungs through the atrium into the ventricle. One would suppose, therefore, that changes in PV’s ostia induced by ablation could influence LA conduit properties.

Moreover, the LA geometry and pressures are strictly related to LV function. It is known that LA conduit contribution to LV stroke volume increases with worsening of LV diastolic function. The conduit flow rate expressed in ml/sec is regarded as a marker of LV relaxation [17]. This parameter increases in exercise and affects an increment in LV filling during early diastole.

We found worsening of both LA and LV parameters during early diastolic phase of the cardiac cycle in the CB subgroup. We observed significant decrease in both novel strain parameter; LAScd and standard tissue Doppler-derived e′ in patients with better preserved baseline values of these parameters. Atrial fibrillation often coexists with LV diastolic dysfunction. Whether our observation of LA conduit dysfunction after ablation will result in clinical symptoms such as deterioration of exercise tolerance warrants further studies.

It has been suggested that CB ablation for PAF may lead to more extensive LA injury than RF ablation. The mechanisms of tissue injury in these two methods of ablation are different. Changes in myocardial tissue in response to cryoenergy include circumscribed fibrotic lesions with less inflammation as compared with RF energy. Cryoablation leads to cellular injury caused by a combination of ice crystal-induced osmotic stress, with subsequent membrane lysis and enzyme inhibition, as well as ischemic cellular necrosis caused by microcirculatory failure [18]. Rewarming during the procedure exacerbates this injury. There are many studies investigating the biochemical myocardial injury markers after ablation; however, the results are conflicting. The majority showed that CB ablation caused more significant myocardial damage as compared with RF ablation [6,8,19,20]. There are studies reporting the opposite [21] or comparable findings [7].

The data on the difference in ablation-induced scar formation by RF and CB techniques are scarce. The subanalysis of the DECAAF II study, with the use of cardiac magnetic resonance (CMR), demonstrated that these two techniques have different effects on the LA post-ablation scar. The CB ablation creates more extensive scarring around PV’s ostia than RF ablation—especially in patients without AF recurrence [22]. 

The elimination of AF with ablation is an unquestionable consequence, but the amount of LA scarring caused by ablation could influence LA structural and functional remodeling. The LA enlargement could be reversed after successful ablation, and both techniques can be effective in LA electrical and structural reverse-remodeling in paroxysmal AF [23]. Similarly, in our study, the LA diameter decreased after ablation and the changes of LA dimension did not differ significantly between the CB and RF subgroups.

The LA function following different ablation strategies had been rarely evaluated. The Cryo-LAEF study prospectively compared the effects of RF and CB ablation on LA function in real-time 3D TTE at 1 and 3 months after the procedure and observed improved or stable LA ejection fraction in both ablation subgroups [24]. Nonetheless, LA strain and strain rates parameters were not assessed in the study. Moreover, in the first 3 months after ablation, recurrences of arrhythmia can be present which may impact on LA function, and longer follow-up duration should be performed for evaluating LA function.

Only small studies estimating effects of different CA strategies on LA function using advanced echocardiographic techniques evaluating LA wall properties have been published. Ling You et al. found that LA strain parameters decreased just after CA and recovered within 1 to 3 months with full recoveries of these parameters to levels similar to baseline at 9 months after CA [19]. Moreover, temporary changes in LA function evaluated by STE were not significantly different among patients who received different CA strategies, including RFCA, CB, and 3D-mapping-guided CB within 1 year after ablation. However, contrary to our study, patients with recurrence of AF were included in the analysis. During and after AF, LA function as a reservoir and conduit is impaired, and systolic function could not exist. It has been shown that LA strain is impaired and reduction of the positive LA strain curve during the reservoir phase is observed in AF [25]. By definition, patients with AF have lower LA strain as an effect of impairment of atrial mechanical function; therefore, LA function measurement may result in lower diagnosis accuracy. To avoid this, we analyzed patients without recurrence of AF after ablation. In our study, the RF ablation subgroup had more frequent use of B-blocker treatment at the baseline. The difference seems to be by chance and is unlikely to be of clinical relevance and may impact on LA evaluation.

The impact of ablation scars on cardiac performance was also studied by biomedical engineering with the use of a four-chamber heart model [26]. There is biomechanical evidence that the position and extent of ablation scars are not only important for the termination of arrhythmia but also determine both atrial and ventricular function.

Moreover, the observations of the biochemical data showed that parameters used in the assessment of the LV function like natriuretic peptides significantly correlated with LA volume parameters [27,28]. The secretion of natriuretic peptides is stimulated by LV pressure and volume overload, which is closely related to the LA geometry, pressures, and function.

There is an important question—whether the overall benefits from maintenance of sinus rhythm compensate the potential adverse effect of LA scarring in patients with PAF treated by ablation. Specific to ablation management, the comprehensive pre- and postprocedural echocardiographic evaluation of LA function that could affect procedural and postprocedural therapy planning should be of special importance. Our study showed that adding LA strain analysis to the routine echocardiographic examination might help to evaluate subclinical dysfunction of LA after CB ablation. Clinical trials are needed to evaluate the potential impact of this finding on clinical outcomes such as deterioration of LV diastolic function, exercise intolerance, or AF recurrence in the future. Moreover, further studies are needed to guide optimal selection of candidates for CB ablation.

## 5. Conclusions

Cryoballoon ablation causes more significant LA dysfunction compared with RF ablation for PAF in patients who underwent a successful procedure and had better preserved baseline LA function. The clinical significance of this finding warrants further investigations.

## 6. Limitations

Firstly, this was a retrospective and single-center study, with follow-up duration limited to one year. However, the echocardiographic analysis was conducted in a prospective manner on previously recorded images by investigators blinded to the mode of ablation.

Secondly, the study group was relatively small. However, the follow-up period was completed in all patients, and the number of patients was sufficient to perform meaningful statistical analysis.

Thirdly, patients were not randomized into CB or RF subgroups at the beginning of this study; however, the choice of ablation method was due to logistical reasons rather than medical selection. Lack of randomization resulted in the baseline differences in proportion of patients treated with beta blockers. The data on beta blockers’ effect on LA function are scarce. We found one study of 212 patients with arterial hypertension, without AF or HF, which revealed that beta blockers’ use was associated with impaired LA reservoir, conduit, and booster pump strain estimated in CMR [29]. In our population, the value of baseline LAS in all LA phases was comparable between RF and CB subgroups. Opposite to the above-mentioned study, we observed higher baseline LASRr in RF comparing to the CB ablation subgroup, in spite of a higher proportion of beta blockers’ use in the RF subgroup. One could assume that the effects of beta blockers on the echocardiographic parameters measured in this study were probably small.

## Figures and Tables

**Table 1:** Baseline clinical characteristic of the study group.

Clinical Characteristic	Study Group N = 90	RF N = 46 (51%)	CB N = 44 (49%)	RF vs. CB *p*
Men *n* (%)	59 (65.6%)	34 (73.9%)	25 (56.8%)	0.09
Age (years)	57.2 ± 9.7	56.2 ± 10.1	59.2 ± 9.3	0.35
BMI (kg/m^2^)	29.0 ± 3.5	29.2 ± 3.0	28.8 ± 4.0	0.63
Duration of AF (years)	3.0 (1.6–8.0)	3.0 (2.0–7.0)	2.0 (1.5–10)	0.39
DM	4 (9.1%)	3 (14.3%)	1 (4.3%)	0.34
CAD	6 (6.7%)	3 (6.5%)	3 (6.8%)	1.00
Arterial hypertension	59 (65.6%)	28 (60.9%)	31 (70.4%)	0.34
Scale CHA2DS2-VASc	1 (1–2)	1 (0–2)	2 (1–2)	0.09
Hyperlipidemia	36 (40%)	20 (43.5%)	16 (36.4%)	0.49
OSA	4 (4.4%)	2 (4.3%)	2 (4.5%)	1.00
Systolic BP (mmHg)	133.1 ± 11.2	133.0 ± 12.4	133.3 ± 9.9	0.90
Diastolic BP (mmHg)	86.4 ± 7.0	85.9 ± 7.2	86.9 ± 6.9	0.47
B-blockers	55 (61.8%)	36 (78.3%)	19 (44.2%)	0.0009
AA	53 (76.8%)	32 (84.2%)	21 (67.7%)	0.11
ACE-I	46 (51.1%)	19 (41.3%)	27 (61.4%)	0.06

Abbreviations: RF—radiofrequency ablation, CB—cryoballoon ablation, BMI—body mass index, AF—atrial fibrillation, DM—diabetes mellitus, CAD—coronary artery disease, OSA—obstructive sleep apnea, BP—blood pressure, AA—antiarrhythmic therapy, ACE-I—angiotensin converting enzyme inhibitor. Values are expressed as the mean ± SD and range or number and (%).

**Table 2:** Echocardiographic parameters after 12 months of follow-up in the whole study group.

Parameter	Baseline	12 Months	Δ 95% CI	*p*
LVEDd (mm)	48.5 ± 4.5	47.3 ± 4.6	−1.2 (−1.8; −0.5)	<0.001
LVEF (%)	63.1 ± 6.9	62.5 ± 3.9	−0.6 (−1.6; 0.4)	0.23
LAd (mm)	38.3 ± 4.1	36.8 ± 3.6	−1.5 (−2.2; −0.9)	<0.001
LAV index (mL/m^2^)	34.0 ± 12.2	32.7 ± 8.4	−1.3 (−3.1; 0.6)	0.17
Mitral E (cm/s)	0.7 ± 0.2	0.7 ± 0.2	0.0 (0.0; 0.1)	0.03
Mitral A (cm/s)	0.6 ± 0.2	0.6 ± 0.2	0.0 (−0.0; 0.5)	0.31
e′ (cm/s)	9.0 ± 1.9	9.4 ± 2.1	0.5 (0.2; 0.8)	0.004
a′ (cm/s)	8.6 ± 2.1	9.0 ± 1.8	0.4 (0.0; 0.7)	0.04
E/e′	7.2 ± 2.3	7.7 ± 1.9	0.0 (−0.4; 0.3)	0.81
LASr (%)	27.8 ± 6.9	27.8 ± 6.2	0.0 (−1.3; 1.1)	0.91
LAScd (%)	15.0 ± 4.8	14.2 ± 4.2	−0.8 (−1.7; 0.1)	0.06
LASct (%)	12.8 ± 4.5	13.6 ± 3.7	0.8 (−0.3; 1.8)	0.14
LASRr (s^−1^)	1.1 ± 0.3	1.3 ± 0.3	0.1 (0.1; 0.2)	<0.001
LASRcd (s^−1^)	1.1 ± 0.3	1.2 ± 0.3	0.1 (0.1; 0.2)	<0.001
LASRct (s^−1^)	1.4 ± 0.5	1.5 ± 0.7	0.0 (−0.0; 0.2)	0.20
LA stiffness	0.3 ± 0.2	0.3 ± 0.1	−0.0 (−0.1; 0.0)	0.27

Abbreviations: LVEDd—left ventricular end diastolic diameter, LVEF—left ventricular ejection fraction, LAd—left atrial diameter, LAV—left atrial volume, LASr—left atrial reservoir strain, LAScd—left atrial conduit strain, LASct—left atrial contractile strain, LASRr—left atrial reservoir strain rate, LASR cd—left atrial conduit strain rate, LASRrct—left atrial contractile strain rate. e′—early; a′—late. Values are expressed as the mean ± SD.

**Table 3:** Comparison of the changes of LA function parameters after 12 months of follow-up regarding the mode of ablation.

Parameter	RF			CB			CB vs. RF	
	Baseline	12 Months	Adj. Δ (95% CI) 12m—Baseline	Baseline	12 Months	Adj. Δ (95% CI) 12m—Baseline	Adj. Δ CB–Adj. Δ RF	*p*
LAd (mm)	38.4 ± 4.0	36.9 ± 3.8	−1.5 (−2.3; −0.7) *	38.1 ± 4.2	36.6 ± 3.3	−1.6 (−2.4; −0.7) *	0.0 (−1.2; 1.1)	0.94
LAV index (mL/m^2^)	34.3 ± 9.9	33.1 ± 8.8	−1.1 (−2.8; 0.7)	33.6 ± 14.6	32.3 ± 8.0	−1.5 (−3.4; 0.4)	−0.4 (−3.0; 2.2)	0.75
Mitral E (cm/s)	0.7 ± 0.2	0.7 ± 0.2	0.0 (0.0; 0.1)	0.7 ± 0.2	0.7 ± 0.1	0.0 (0.0; 0.1)	0.00 (−0.1; 0.1)	0.99
Mitral A (cm/s)	0.6 ± 0.2	0.6 ± 0.2	0.0 (0.0; 0.0)	0.6 ± 0.2	0.6 ± 0.1	0.0 (0.0; 0.1)	0.1 (0.0; 0.10)	0.07
e′ (cm/s)	8.9 ± 2.1	9.5 ± 2.5	0.6 (0.2; 1.1)	9.1 ± 1.7	9.3 ± 1.7	0.3 (−0.2; 0.7)	−0.4 (−1.0; 0.2)	0.23
a′ (cm/s)	8.8 ± 1.8	9.1 ± 1.6	0.3 (−0.1; 0.7)	8.4 ± 2.3	8.9 ± 2.0	0.5 (0.0; 0.5) *	0.1 (−0.5; 0.7)	0.67
E/e′	7.8 ± 2.4	7.6 ± 2.1	−0.1 (−0.5; 0.3)	7.7 ± 2.2	7.7 ± 1.8	0.0 (−0.4; 0.5)	0.2 (−0.5; 0.8)	0.62
LASr (%)	27.8 ± 6.7	28.0 ± 6.1	0.2 (−1.3; 1.6)	27.8 ± 7.4	27.5 ± 6.4	−0.3 (−1.9; 1.2)	−0.5 (−2.6; 1.6)	0.65
LAScd (%)	14.7 ± 4.1	14.6 ± 4.8	−0.2 (−1.2; 0.8)	15.3 ± 5.4	13.7 ± 3.4	0.26 (−0.18; 0.70)	−1.3 (−2.8; 0.2)	0.09
LASct (%)	13.1 ± 4.9	13.4 ± 3.4	0.5 (−0.6; 1.5)	12.5 ± 3.9	13.8 ± 4.1	1.1 (−0.0; 2.3)	0.7 (−0.9; 2.3)	0.40
LASRr (s^−1^)	1.2 ± 0.22	1.3 ± 0.31	0.1 (0.0; 0.2) *	1.1 ± 0.3	1.2 ± 0.3	0.1 (0.1; 0.2) *	0.0 (0.1; 0.2)	0.52
LASRcd (s^−1^)	1.1 ± 0.3	1.3 ± 0.4	0.2 (0.1; 0.2) *	1.1 ± 0.3	1.2 ± 0.3	0.1 (0.0; 0.2) *	0.0 (−0.2; 0.1)	0.63
LASRct (s^−1^)	1.5 ± 0.4	1.5 ± 0.3	0.1 (0.0; 0.2)	1.4 ± 0.5	1.5 ± 0.4	0.0 (−0.1; 0.2)	0.0 (−0.2; 0.1)	0.60
LA stiffness	0.3 ± 0.2	0.3 ± 0.1	0.0 (−0.1; 0.0)	0.3 ± 0.2	0.3 ± 0.1	0.0 (−0.1; 0.0)	0.0 (0.0; 0.1)	0.68

*—*p* < 0.05; *p* for Δ adjusted for baseline values. Abbreviations: RF—radiofrequency ablation, CB—cryoballoon ablation, LAd—left atrial diameter, LAV—left atrial volume, LASr—left atrial reservoir strain, LAScd—left atrial conduit strain, LASct—left atrial contractile strain, LASRr—left atrial reservoir strain rate, LASR cd—left atrial conduit strain rate, LASRrct—left atrial contractile strain rate. Values are expressed as the mean ± SD or mean [range] wherever applicable.

**Table 4:** Comparison of the changes of LA function parameters, for which the difference between RF and CB subgroups depended on the baseline values.

Parameter	RF		CB		CB vs. RF	
	Baseline Tested Values	Adj. Δ [95% CI] 12m—Baseline	Baseline Tested Values	Adj. Δ [95% CI] 12m—Baseline	Adj. Δ CB—Adj. Δ RF	*p*
e′ (cm/s)	5 8 12	0.7 (−0.2; 1.5) 0.7 (0.2; 1.1) * 0.6 (−0.1; 1.3)	5 8 12	2.1 (1.0; 3.2) * 0.7 (0.2; 1.2) * −1.1 (−1.9; −0.2) *	1.4 (0.0; 2.8) 0.1 (−0.6; 0.7) −1.7 (−2.8; −0.6) *	0.05 0.83 0.003
LAScd (%)	8 16 28	0.8 (−1.0; 2.6) −0.3 (−1.3; 0.7) −1.9 (−5.1; 1.4)	8 16 28	2.9 (1.1; 4.6) * −2.1 (−3.1; −1.1) * −9.5 (−12.0; −6.9) *	2.1 (−0.5; 4.5) −1.8 (−3.2; −0.4) * −7.6 (−11.7; −3.4) *	0.11 0.014 <0.001

*—*p* < 0.05; *p* for Δ adjusted for baseline values. Abbreviations: RF—radiofrequency ablation, CB—cryoballoon ablation, LAScd—left atrial conduit strain. Values are expressed as the mean ± SD or mean [range] wherever applicable.

## Data Availability

Not applicable.
